# Clinical factors and major pathological response after neoadjuvant chemoimmunotherapy in potentially resectable lung squamous cell carcinoma

**DOI:** 10.3389/fonc.2024.1265228

**Published:** 2024-04-12

**Authors:** Ye Wang, Yingqiu Song, Runze Wang, Yu Wu, Mo Li, Ke Xu, Rong He, Zheng Wang, Qingqing Li, Feng-Ming (Spring) Kong, Tianlu Wang

**Affiliations:** ^1^ Department of Radiotherapy, Cancer Hospital of China Medical University, Liaoning Cancer Hospital & Institute, Cancer Hospital of Dalian University of Technology, Shenyang, Liaoning, China; ^2^ School of Graduate, Dalian Medical University, Dalian, China; ^3^ Department of Breast Surgery, Liaoning Cancer Hospital and Institute, Shenyang, Liaoning, China; ^4^ Department of Thoracic Surgery, Liaoning Cancer Hospital and Institute, Shenyang, Liaoning, China; ^5^ Department of Endoscopy, Liaoning Cancer Hospital and Institute, Shenyang, Liaoning, China; ^6^ Department of Clinical Oncology, The University of Hong Kong-Shenzhen Hospital, Shenzhen, China; ^7^ Department of Clinical Oncology, Li Ka Shing Faculty of Medicine, The University of Hong Kong, Hong Kong, Hong Kong SAR, China; ^8^ Faculty of Medicine, Dalian University of Technology, Dalian, China

**Keywords:** lung squamous cell carcinoma, neoadjuvant chemoimmunotherapy, major pathologic response, nomogram, biomarkers

## Abstract

**Objective:**

Major pathological response (MPR) helps evaluate the prognosis of patients with lung squamous cell carcinoma (LUSC). However, the clinical factors that affect the achievement of MPR after neoadjuvant chemoimmunotherapy (NCIO) in patients with LUSC remain unclear. This study aimed to explore the clinical factors affecting the MPR after NCIO in patients with potentially resectable LUSC.

**Methods:**

This retrospective study included patients with stage IIB-IIIC LUSC who underwent surgical resection after receiving NCIO at a center between March 2020 and November 2022. In addition to the postoperative pathological remission rate, sex, age, body mass index (BMI), smoking history, TNM stage, hematological and imaging test results, and other indicators were examined before NCIO. According to the pathological response rate of the surgically removed tumor tissue, the patients were split into MPR and non-MPR groups.

**Results:**

In total, 91 LUSC patients who met the study’s eligibility criteria were enrolled: 32 (35%) patients in the non-MPR group and 59 (65%) in the MPR group, which included 43 cases of pathological complete remission (pCR). Pre-treatment lymphocyte level (LY) (odds ratio [OR] =5.997), tumor burden (OR=0.958), N classification (OR=15.915), radiographic response (OR=11.590), pulmonary atelectasis (OR=5.413), and PD-L1 expression (OR=1.028) were independently associated with MPR (all P < 0.05). Based on these six independent predictors, we developed a nomogram model of prediction having an area under the curve (AUC) of 0.914 that is simple to apply clinically to predict the MPR. The MPR group showed greater disease-free survival (DFS) than the non-MPR group, according to the survival analysis (P < 0.001).

**Conclusion:**

The MPR rate of NCIO for potentially resectable LUSC was 65%. LY, tumor burden, N classification, radiographic response, pulmonary atelectasis, and PD-L1 expression in patients with LUSC before NCIO were the independent and ideal predictors of MPR. The developed nomogram demonstrated a good degree of accuracy and resilience in predicting the MPR following NCIO, indicating that it is a useful tool for assuring customized therapy for patients with possibly resectable LUSC.

## Introduction

Lung cancer mortality and incidence rates have risen in recent years, posing a serious hazard to human health (1). Only about 20–25% of patients with non-small cell lung cancer (NSCLC) can undergo surgical tumor removal (2), and up to 30–55% of those who undergo radical surgery experience relapse and eventually die from the disease (3, 4). Even adjuvant or neoadjuvant chemoradiotherapy can only marginally improve survival by 5% (5, 6). Because of its unique clinicopathologic characteristics, such as advanced age, advanced disease at diagnosis, comorbidities, a propensity to invade large blood vessels, and central tumor location, lung squamous cell carcinoma (LUSC), which accounts for 25–30% of lung cancers, frequently makes surgical resection difficult. Furthermore, the median survival of patients with LUSC is 30% shorter than that of patients with other NSCLC subtypes (7). Additionally, common driver mutations occur in less than 7% of LUSC cases (8–10), rendering the affected patients ineligible for targeted therapy (11). Nevertheless, immunotherapy has proven to be an effective treatment option for people with LUSC (12), particularly preoperative neoadjuvant chemoimmunotherapy (NCIO), which has demonstrated promising efficacy (13, 14).

Previous classic trials of neoadjuvant therapy for NSCLC, such as the CheckMate-159, NEOSTAR, CheckMate-816, NADIM, and NCT02716038, and many other studies have demonstrated that NCIO has considerable advantages in terms of the short-term outcomes, such as security, tolerability, and major pathologic response (MPR) (13–17). Currently, NCIO is the most important treatment modality, and there may be some synergy between the two components. Chemotherapy can induce mutations in tumor cells, resulting in new epitopes that enhance tumor immunogenicity, fully activate the immune response, and improve the efficacy of immunotherapy (18, 19). Immunotherapy may remove the residual lesions or small metastases to achieve tumor regression, increase the R0 resection ratio, and eliminate micrometastases, ultimately improving patients’ overall survival (20). Previous studies have indicated that patients who achieve MPR or pathological complete remission (pCR) after treatment demonstrate better survival in terms of progression-free survival (PFS), disease-free survival (DFS), and overall survival (OS). Therefore, MPR is often used as a surrogate endpoint (14, 21).

However, only a fraction of patients can achieve MPR and benefit from NCIO. Currently, data on the clinical indicators affecting MPR achievement, including hematological indicators, are incomplete. Therefore, we conducted this study to identify validated signatures to determine patient subgroups that might benefit from NCIO.

It is well known that the incidence of EGFR mutations is higher in Asian patients with lung adenocarcinoma (LUAD), and the overall EGFR mutation prevalence in the mainland Chinese population is 50.2% (22). In addition, several clinical studies (23–25) have shown the poor efficacy of immunotherapy for NSCLC with EGFR mutations; therefore, we temporarily excluded patients with LUAD from this study. This study aimed to explore the clinical factors affecting the achievement of MPR in patients with potentially resectable LUSC undergoing NCIO and develop a nomogram prediction model for selecting patients with potentially resectable (IIB-IIIC) LUSC who can achieve MPR after NCIO, which can provide a foundation for guiding the individualized and accurate treatment of patients with potentially resectable LUSC.

## Materials and methods

### Study design and patients

**Figure 1:**
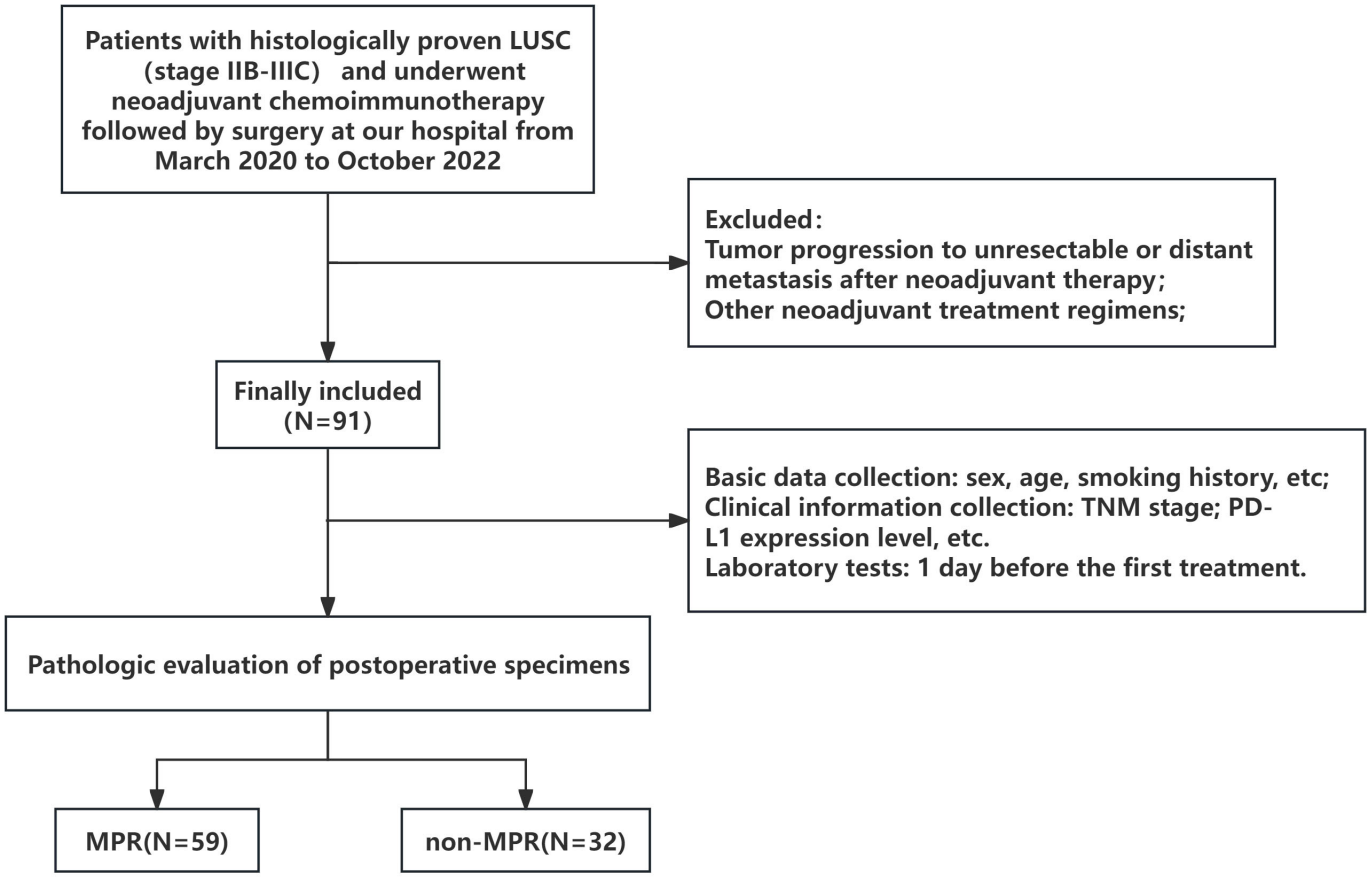
Patient selection flowchart.

From March 2020 to October 2022, we conducted a retrospective analysis of the clinical data of patients with LUSC who received successful surgical resection following NCIO at Liaoning Cancer Hospital. The following were the inclusion criteria (1): aged 18 years or older (2), pathologically demonstrated LUSC (3), clinical stage IIB-IIIC at initial diagnosis in accordance with the tumor staging (8th edition) of the American Joint Committee on Cancer (4), no other prior systemic antitumor therapy (5), Eastern Cooperative Oncology Group (ECOG) performance status (PS) ≤2 (6), lung cancer that was amenable to resection at the time of the initial multidisciplinary diagnostic and treatment (MDT) evaluation (7), received NCIO before resection, and (8) complete clinical data available, including imaging and pathology data before and after treatment. The following were the exclusion criteria (1): at the second MDT evaluation, the tumor had developed into unresectable disease or distant metastasis following neoadjuvant therapy (2); contraindications to immunotherapy; and (3) a history of additional malignant tumors during the previous 5 years. The flow chart of the study is shown in **Figure 1**.

### Data collection

Basic patient data, such as sex, age, body mass index (BMI), and smoking history, were recorded. Clinical information was also collected, including the pathology type, TNM stage, PD-L1 expression level, and pre-treatment tumor burden (defined as the long diameter × the short diameter of the largest diameter plane, as assessed by enhanced computed tomography [CT]). Laboratory tests, including those for leukocytes, hemoglobin, platelets, tumor markers, albumin, and lactate dehydrogenase, were performed 1 day before the first treatment. After NCIO, a second enhanced CT examination of the lung was performed to assess the efficacy.

### Laboratory tests methods

Fasting venous blood was drawn from all enrolled patients at Liaoning Cancer Hospital on the day before the first treatment. Blood samples were stored at 4°C for no more than 2 hours before analysis. Red blood cell count, white blood cell count, hemoglobin concentration, and platelet count were tested using a blood analyzer with a special kit. Liver function, kidney function, and the cardiac enzyme profile were assessed using enzymatic methods. Tumor markers were detected using a chemiluminescence immunoassay. All data are reported according to the international system of units.

### Treatment protocol

Patients received the following drugs intravenously: pembrolizumab (200 mg), tislelizumab (200 mg), sintilimab (200 mg), camrelizumab (200 mg), nivolumab (360 mg), toripalimab (240 mg), paclitaxel (135-175 mg/m^2^), paclitaxel liposome (135-175 mg/m^2^), nab-paclitaxel (260 mg/m^2^), docetaxel (60-75 mg/m^2^), gemcitabine (1000 mg/m^2^, d1, d8), carboplatin (area under the curve [AUC], 5; 5 mg/ml per min), and cisplatin (80-100 mg/m^2^), on day 1 of each 21-day cycle with a total of one to five cycles.

### Outcomes

The main study outcome was the MPR rate, which combined the pCR and MPR. pCR was defined as the lack of viable tumor cells in both the primary and metastatic lymph nodes. In contrast, MPR was defined as ≤10% of viable tumor cells in the original tumor bed, regardless of whether viable tumor cells were present in the metastatic lymph nodes (26). The Response Evaluation Criteria in Solid Tumors (RECIST) v1.1 recommendations were used to evaluate the radiographic response (27). The radiographic response was divided into four categories: complete response (CR), partial response (PR), stable disease (SD), and progressive disease (PD). CR was defined as the disappearance of all NSCLC tumor lesions, which was maintained for 4 weeks; PR was defined as a reduction in the NSCLC lesions by 30% or more, which was maintained for 4 weeks; SD was defined as a reduction in the NSCLC lesions by 30% or less, or no increase, which was maintained for 4 weeks; PD was defined as a 20% or greater increase in diameter, a relative rise in the target lesions, or the emergence of one or more additional lesions. The period from the surgery date until the diagnosis of recurrence/metastasis, death, or last follow-up was referred to as disease-free survival (DFS).

### Statistical analyses

An independent samples t-test was used to compare two sets of continuous variables with a normal distribution and homogeneous variance reported as means ± standard deviation. The Mann-Whitney U test was used to compare the medians (interquartile range [IQR]) of two groups of continuous statistics with abnormal distributions or variance heterogeneity. The medians of the two groups were given (interquartile range [IQR]). The chi-square or Fisher’s exact tests compared classification variables reported as percentages (%). Odds ratios (ORs) were calculated using univariate logistic regression analyses. A multivariate binary logistic model was applied for regression analyses, and factors with P < 0.05 in the univariate logistic regression analyses were incorporated into the multivariate analyses, with the calculation of the OR and 95% confidence interval (CI). The Kaplan–Meier method was used to create survival curves, and the log-rank test assessed the differences between the survival curves. Statistical analyses were performed using IBM SPSS (version 26.0; IBM Corporation, Armonk, NY) software. The pROC package created receiver operating characteristic (ROC) curves and calculated the area under the curve (AUC) values. The Rms package of R4.2.1 was used to create a nomogram and calibration curve and perform cross-validation. The threshold for statistical significance was P < 0.05.

## Results

### Patient characteristics

A total of 91 eligible patients with LUSC between March 2020 and October 2022 were included in this study. Follow-up was carried out by telephone, outpatient check-up, and hospital admission until January 31, 2023. According to the pathological response rate of the surgically removed tumor tissue, the patients were split into the MPR and non-MPR groups. Fifty-nine patients received NCIO and achieved MPR (among them, 43 patients achieved pCR) with an MPR rate of 64.8%.

Of the 91 patients enrolled, the mean age was 59.49 ± 6.98 years; range 44–77 years. The male to female ratio was 84:7 (men, 92.3%; women, 7.7%); 69 patients (75.9%) had a history of smoking; and 51 patients (56.0%) had stage IIB–IIIA LUSC. Only 1 of the 91 patients had a R1 resection at the time of surgery, and the remaining 90 patients had R0 resections. The mean total number of lymph nodes dissected was 22.63 ± 11.06 (**Table 1**).

**Table 1:** General clinical characteristics were associated with major pathological response (MPR).

Characteristics	All patients (n = 91)	Non-MPR(N=32)	MPR(N=59)	X^2^/T	*P* value
**Age (years)**	59.49 ± 6.98	57.88 ± 7.13	60.37 ± 6.79	-1.646	0.103
Sex					
Male	84(92.3%)	30	54	0.145	0.704
Female	7(7.7%)	2	5		
**BMI**	24.46 ± 3.26	25.30 ± 2.47	24.01 ± 3.56	1.816	0.073
Smoking					
No	22(24.1%)	9	13	0.420	0.517
Yes	69(75.9%)	23	46		
T classification					
T1-2	36(39.6%)	11	25	0.555	0.456
T3-4	55(60.4%)	21	34		
N classification					
N0-1	28(30.8%)	5	23	5.314	**0.021**
N2-3	63(69.2%)	27	36		
Clinical stage					
IIB-IIIA	51(56.0%)	15	36	1.684	0.194
IIIB-IIIC	40(44.0%)	17	23		
Radiographic response					
SD	30(32.9%)	21	9	23.820	**<0.001**
PR	61(67.1%)	11	50		
Pulmonary atelectasis					
No	53(58.3%)	14	39	4.262	**0.039**
Yes	38(41.7%)	18	20		
**Tumor burden(cm^2^)**	26.19 ± 18.41	34.79 ± 18.28	21.53 ± 16.88	3.475	**0.001**
**PD-L1 expression(%)**	55.0(10.0-81.0)	23.50(3.50-62.25)	65(30.0-85.0)	-2.985	**0.003**
Thoracic adhesion					
No	43(47.3%)	16	27	0.149	0.699
Yes	48(52.7%)	16	32		
Treatment cycles					
≤2 cycles	56(61.5%)	21	35	0.348	0.555
≥3 cycles	35(38.5%)	11	24		
Immunotherapy regimens					
Tislelizumab	41(45.0%)	16	25	0.556	0.907
Sintilimab	30(33.0%)	10	20		
Camrelizumab	13(14.3%)	4	9		
Others	7(7.7%)	2	5		
Chemotherapy regimens					
Nab-paclitaxel–based	71(78.0%)	25	46	1.172	0.557
Gemcitabine-based	11(12.1%)	5	6		
Others	9(9.9%)	2	7		
Type of surgery					
Video-assisted thoracoscopic surgery	60(65.9%)	22	38	0.174	0.676
Thoracotomy	31(34.1%)	10	21		
**Number of lymph nodes dissected**	22.63 ± 11.06	25.31 ± 11.96	21.17 ± 10.36	1.725	0.088

SD, stable disease; PR, partial response; PD-L1, programmed death receptor-1 ligand; IQR, interquartile range; MPR, major pathologic response.

Bold text represents variables with statistical significance.

Basic patient and tumor characteristics are shown in **Table 1**. A comparative analysis of characteristics that may have affected the achievement of MPR, including sex, age at onset, smoking history, TNM classification, pre-treatment PD-L1 expression level, pre-treatment tumor burden (product of long and short tumor diameters), and laboratory test results, was performed in both groups (**Table 1**).

### General clinical characteristics associated with MPR achievement after NCIO

**Figure 2:**
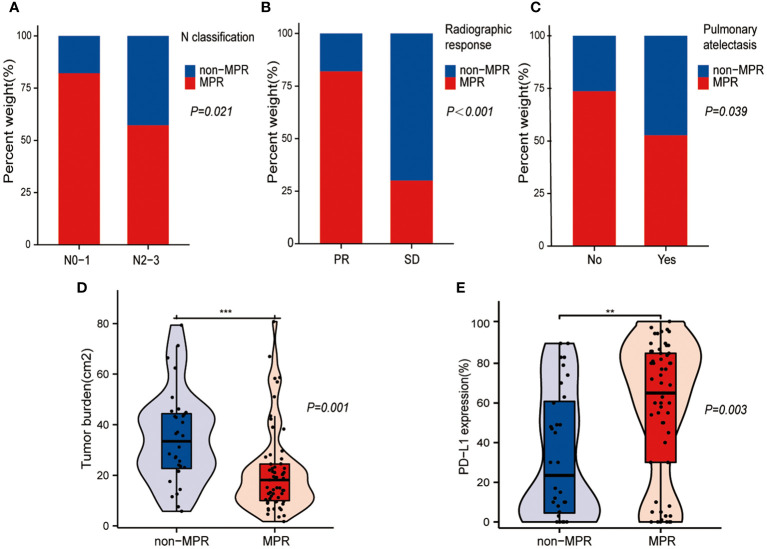
Statistically different general clinical characteristics between the major pathological response (MPR) and non-MPR groups. **(A)** Bar plot shows that higher MPR rates in patients with earlier N classification. **(B)** Bar plot shows that higher MPR rates in patients with partial response (PR). **(C)** Bar plot shows that higher MPR rates in patients without pulmonary atelectasis. **(D)** Box plot shows that the MPR group had a smaller tumor burden. **(E)** Box plot shows that the MPR group had a higher pre-treatment PD- L1 expression level in the tumor tissue. **: p < 0.01, ***: p < 0.001.

**Figure 2** shows the statistically significant differences in the general clinical characteristics between the non-MPR and MPR groups after treatment with NCIO. The results show a higher rate of MPR achievement following treatment with NCIO in patients with the following characteristics: an earlier N classification (**Figure 2A**, P = 0.021), a radiographic response of PR (**Figure 2B**, P < 0.001), a smaller tumor burden (**Figure 2D**, P = 0.001), and a higher pre-treatment tumor tissue PD-L1 expression level (**Figure 2E**, P = 0.003), additionally, those without obstructive pneumonia/atelectasis (**Figure 2C**, P = 0.039). Age, sex, BMI, smoking history, T classification, clinical stage, presence of thoracic adhesions during surgery, and number of NCIO cycles were not significant different between the MPR and non-MPR groups (P>0.05) (**Table 1**).

### Laboratory characteristics associated with MPR achievement after NCIO

**Figure 3:**
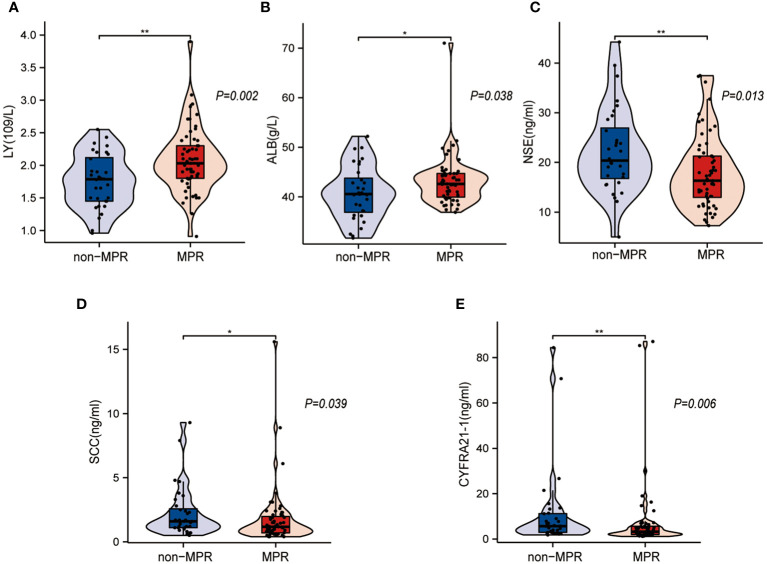
Statistically different laboratory characteristics between the major pathological response (MPR) and non-MPR groups. **(A, B)** Box plots show that the MPR group had a higher lymphocyte count (LY) and albumin (ALB) level. **(C–E)** Box plots show that the non-MPR group had higher levels of neuron-specific enolase (NSE), squamous cell carcinoma antigen (SCC), and cytokeratin-19 (CYFRA21-1). *: p < 0.05, **: p < 0.01.

**Figure 3** illustrates the laboratory characteristics that were significantly different between the non-MPR and MPR groups. The results show that the lymphocyte count (LY) (**Figure 3A**, P=0.002) and albumin (ALB) (**Figure 3B**, P=0.038) levels were higher in the MPR group than in the non-MPR group, and the difference was statistically significant. The levels of tumor markers, including neuron-specific enolase (NSE), squamous cell carcinoma antigen (SCC), and cytokeratin-19 (CYFRA21-1), were higher in the non-MPR group, and the difference was statistically meaningful (**Figures 3C–E**, P < 0.05). Other blood indicators, including the white blood cell count (WBC), neutrophil count (NE), monocyte count (Mono), hemoglobin level (Hb), platelet count (PLT), lactate dehydrogenase level (LDH), precursor albumin level (pre-ALB), and tumor marker level (carcinoembryonic antigen, CEA), were not significantly different between the groups (P>0.05) (**Table 2**).

**Table 2:** Laboratory characteristics were associated with the major pathological response (MPR).

Characteristics (normal reference value)	All patients (n = 91)	Non-MPR(N=32)	MPR(N=59)	T/Z	P
WBC [3.5-9.5(10^9^/L)]	7.27(6.23-8.79)	7.01(6.13-8.77)	7.48(6.27-8.80)	-0.707	0.480
NE [1.8-6.3(10^9^/L)]	4.63(3.74-6.09)	4.51(3.78-6.01)	4.63(3.70-6.09)	-0.037	0.970
LY [1.1-3.2(10^9^/L)]	1.91(1.65-2.24)	1.79(1.45-2.12)	2.03(1.80-2.30)	-3.060	**0.002**
Mono [0.1-0.6(10^9^/L)]	0.50 ± 0.20	0.52 ± 0.24	0.49 ± 0.17	0.738	0.463
Hb [115-150(g/L)]	139.35 ± 22.12	136.42 ± 29.03	140.93 ± 17.37	-0.928	0.356
PLT [125-350(10^9^/L)]	263.95 ± 69.42	262.09 ± 69.49	264.95 ± 69.96	-0.186	0.853
LDH [109-245(U/L)]	199.00(173.00-241.00)	207.00(167.75-150.50)	198.00(175.00-231.00)	0.611	0.541
Pre-ALB [200-400(mg/L)]	244.09 ± 71.84	234.44 ± 81.07	249.41 ± 66.35	-0.946	0.347
ALB [35-55(g/L)]	42.31 ± 5.22	40.77 ± 5.42	43.14 ± 4.96	-2.107	**0.038**
NSE [0.0-16.3(ng/ml)]	19.52 ± 8.03	22.35 ± 8.54	17.99 ± 7.37	2.548	**0.013**
CEA [0.0-16.3(ng/ml)]	2.70(1.65-4.37)	2.65(1.54-4.26)	2.87(1.69-4.38)	-0.266	0.790
CYFRA21-1 [0.0-16.3(ng/ml)]	3.88(2.12-7.01)	5.68(2.92-11.31)	3.22(1.96-5.72)	2.735	**0.006**
SCC [0.0-16.3(ng/ml)]	1.28(0.80-2.29)	1.60(1.10-2.73)	1.17(0.70-2.04)	2.067	**0.039**

Bold text represents variables with statistical significance.

### Univariable and multivariable analyses identifying independent predictors of MPR

We further calculated the OR using univariate logistic analysis for the significant variables mentioned above. The results showed that only CYFRA21-1 (P = 0.289) and SCC (P = 0.324) were not statistically significant, whereas all other variables were significant (all P < 0.05) (**Table 3**). Further multifactorial regression analysis was performed by combining the results of the univariate analyses. All relevant variables with P < 0.05 in the univariate logistic regression analyses were added as the independent variables. MPR was used as the dependent variable for the multifactor binary logistic regression analyses. The outcomes revealed that LY (P = 0.027), tumor burden (P = 0.033), N classification (P = 0.006), radiographic response (P < 0.001), pulmonary atelectasis (P = 0.022), and pre-treatment PD-L1 expression levels (P = 0.013) were independent elements influencing MPR achievement following NCIO in patients with potentially resectable LUSC (**Table 3**).

**Table 3:** Univariate and Multifactorial binary logistics regression analysis of the major pathological response (MPR) after potentially resectable lung squamous cell carcinoma (LUSC) treated with neoadjuvant chemoimmunotherapy (NCIO).

Characteristics	Univariate logistic regression analysis		Multifactorial binary logistics regression analysis			
	MPR vs.non-MPR		MPR vs.non-MPR			
	Odds Ratio (95% CI)	P value	*β*	OR	Odds Ratio (95% CI)	*P* value
LY(10^9^/L)	5.396 (1.730 - 16.835)	**0.004**	1.791	5.997	1.221 - 29.457	**0.027**
ALB(g/L)	1.119 (1.005 - 1.245)	**0.040**	-0.079	0.924	0.799 - 1.070	0.292
NSE(ng/ml)	0.933 (0.882 - 0.988)	**0.017**	-0.024	0.977	0.904 - 1.055	0.547
CYFRA21-1	0.986 (0.962 - 1.012)	0.289	–	–	–	–
SCC(ng/ml)	0.907 (0.746 - 1.102)	0.324	–	–	–	–
Tumor burden (cm^2^)	0.960 (0.935 - 0.985)	**0.002**	-0.043	0.958	0.921 - 0.996	**0.033**
N classification (N0-1 vs. N2-3)	3.450(1.162-10.243)	**0.026**	2.767	15.915	2.227 - 113.719	**0.006**
Radiographic response (PR vs.SD)	10.606(3.833-29.346)	**<0.001**	2.450	11.590	2.932 - 45.808	**<0.001**
Pulmonary atelectasis (No vs.Yes)	2.507(1.038-6.058)	**0.041**	1.689	5.413	1.273-23.008	**0.022**
PD-L1 expression (%)	1.020(1.007-1.034)	**0.003**	0.028	1.028	1.006 - 1.051	**0.013**

Bold text represents variables with statistical significance.

### Building the clinical prediction model

**Figure 4:**
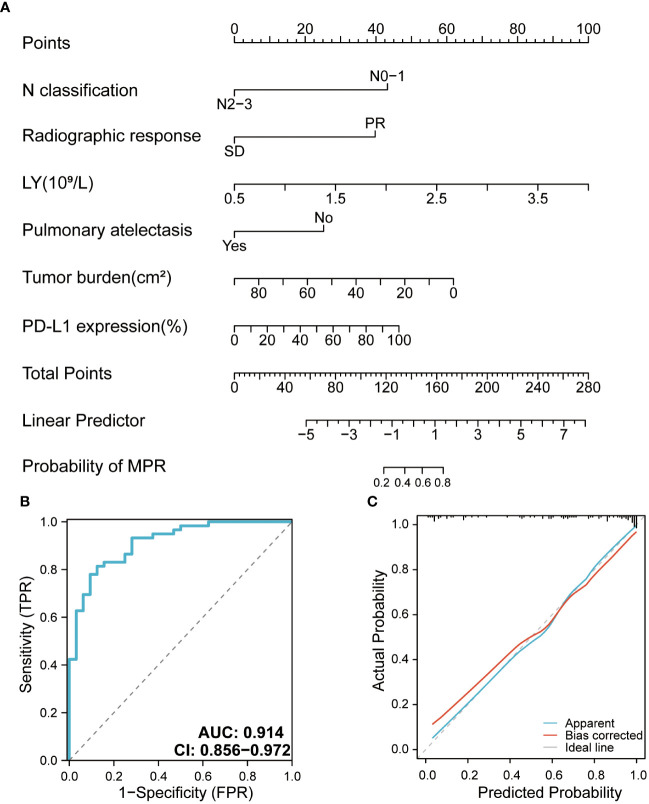
**(A)** Nomogram for the major pathologic response (MPR) predictive model. **(B)** Receiver operating characteristic curve of the MPR model. **(C)** Calibration curve of the MPR model. AUC, area under curve.

The model included six independent influencing factors (LY, tumor burden, N classification, radiographic response, pulmonary atelectasis, and pre-treatment PD-L1 expression level), based on the results of the univariate and multivariate logistic regression analyses. In the nomogram, the vertical line of the score axis was drawn upward according to the actual state of each variable, and a value was assigned to each variable, after which the scores of each variable were added to determine the MPR probability (**Figure 4A**). The AUC of the model was 0.914, which was satisfactory (**Figure 4B**). The calibration curve showed that the appearance and deviation corrected curves were close to the optimal curve (**Figure 4C**). The validation model obtained by combining the ROC with the internal validation curve was more effective than the prediction model.

### Survival analyses

**Figure 5:**
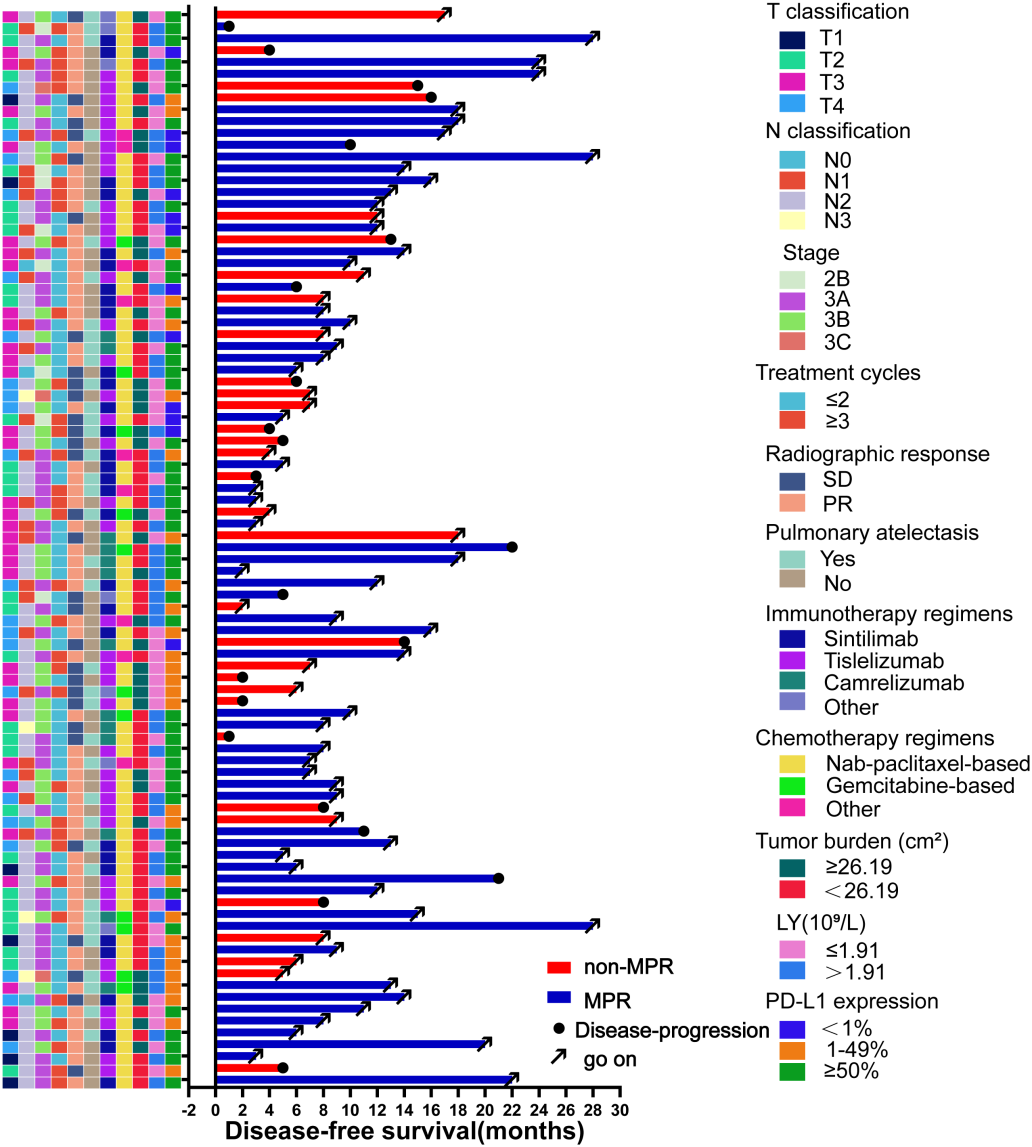
Swimming plot of disease-free survival in the patients who underwent neoadjuvant chemoimmunotherapy (NCIO) (N=91). Each bar represents one patient. The left column shows clinical characteristics. The date cutoff was January 2023; 22 patients who underwent NCIO had disease progression.

**Figure 6:**
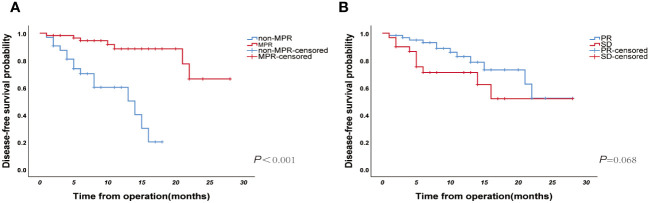
**(A)** The Kaplan–Meier curve of disease-free survival for the major pathological response (MPR) and non-MPR groups (N=91, p<0.001); **(B)** Kaplan–Meier curve of disease-free survival for PR and SD groups (N=91, p=0.068).

Until January 2023, the median follow-up duration was 11 (IQR: 8-17) months. Twenty-two patients showed disease progression during the follow-up period (15 in the non-MPR group and 7 in the MPR group), and the relevant clinical features and follow-up results are shown in **Figure 5**. The overall median DFS was 22 months for all patients, with a median DFS of 14 months in the non-MPR group, whereas the median DFS was not reached in the MPR group. The Kaplan–Meier survival curve showed that the MPR group had a longer DFS and better prognosis than the non-MPR group (**Figure 6A**, P <0.001). Additionally, we analyzed the survival of the PR and SD groups. We found no statistically significant difference between the groups (**Figure 6B**, P = 0.068).

## Discussion

With the increased clinical application of NCIO for NSCLC, pCR and MPR are now internationally recognized as important predictors of treatment efficacy compared to the commonly studied endpoints of PFS and OS, which need longer follow-up times (28). Patients with pCR and MPR have better tumor prognosis and improved OS (29–31). Patients who achieved MPR were found to have increased 5-year OS from 40% to 85% (32, 33).

NCIO exhibits better outcomes than other presurgical neoadjuvant modalities. The CheckMate 159 trial was the first study to apply an immune checkpoint inhibitor (ICI) as a neoadjuvant treatment option for resectable NSCLC, showing an MPR of 45% and pCR of 15% (34). In the first phase III trial to be published, CheckMate 816, neoadjuvant nivolumab with chemotherapy significantly enhanced the MPR rate (36.9% vs. 8.9%, p < 0.05) and pCR rate (24.0% vs. 2.2%, p < 0.001) in patients with NSCLC compared to neoadjuvant chemotherapy alone (16). In this study, the application of NCIO achieved MPR of 65%, which is satisfactory. Most large clinical studies on neoadjuvant targeted therapies also reported MPR rates mostly in the range of 7.7–24.2% (35, 36). These data suggest that NCIO is a novel treatment strategy for resectable NSCLC.

In several previous phase III clinical trials, a certain percentage of patients canceled surgery after receiving NCIO, with disease progression being the primary reason. Considering the possible risks of NCIO, accurately identifying populations suitable for neoadjuvant immunotherapy is critical.

Previous studies have indicated that multiple factors were associated with MPR achievement after NCIO, including peripheral blood inflammatory biomarkers, 18fluorine-fluorodeoxyglucose uptake, PD-L1 expression, tumor mutational burden, and tumor regression rate (37–40). Our study analyzed general clinical and laboratory characteristics and found that LY, tumor burden, N classification, radiographic response, pulmonary atelectasis, and pre-treatment PD-L1 expression level were independent factors affecting MPR rate. Notably, this is the first study to report two new indicators: tumor burden and pulmonary atelectasis. In addition, we developed a predictive model for MPR with an AUC of 0.914. Due to the limited sample size, this study adopted repeated sampling for internal validation, but the model still needs external verification for its scalability. In the future, we may seek collaboration with other research institutions or conduct multicenter studies to evaluate the performance and applicability of the nominated figures across different populations. A comparative survival analysis revealed that grouping by MPR was a better predictor of DFS than grouping by the radiographic response.

In this retrospective study, the efficacy of applying the RECIST guidelines was correlated with the pathological remission of the resected tumor tissue or MPR after surgery. However, the findings of previous studies regarding the correlation between the two are controversial. Studies such as NADIM (17), Checkmate-159 (13), and LCMC3 (41) have shown no significant association between radiographic response and pathological remission. However, one study showed a remarkable correlation between the MPR and objective remission rate (ORR) for the RECIST guidelines (P=0.002) (14). Similarly, the ORR rate was notably higher in the MPR group than that in the non-MPR group in the NEOSTAR trial (P<0.001) (15). Furthermore, survival analysis revealed that there was no significant difference in DFS between the PR and SD groups (*P*=0.068). However, there was a trend towards survival differences and might be due to the relatively short follow-up times (median follow-up, 11 months). Further attention could be paid to this aspect of the difference in subsequent studies.

Increasing clinical and preclinical evidence supports the negative correlation between tumor burden and ICI efficacy (42). In this study, we assessed the tumor burden using enhanced CT. Tumor burden was defined as the longest diameter × the shortest diameter in the maximum diameter plane. Our findings suggest that tumor burden has an independent influence on MPR and that NCIO has a poorer efficacy in patients with a greater tumor burden. As far as we know, this is the first study to report the association between tumor burden and NCIO in LUSC patients. Similarly, a meta-analysis showed that among patients with immuno-monotherapy, a large tumor burden was an independent prognostic factor for worse OS and PFS (43). Related studies have shown that a high tumor burden has a detrimental effect on the efficacy of ICIs (44, 45), directly influencing the host immune system’s capacity to mount successful natural or immunotherapy-induced immunological responses (46). The fact that patients with a high tumor burden exhibited poorer outcomes with NCIO may imply that a different treatment approach is needed for high-burden tumors (e.g., intensive therapy). Therefore, tumor burden assessment should be included in the design of future clinical trials involving ICIs.

The neutrophil-to-lymphocyte ratio (NLR), a blood-based biomarker utilized for various tumor types treated with ICIs, has received much research attention (47). In lung cancer, a high NLR is linked to a poor prognosis (48, 49). However, it consists of two separate biological components: neutrophilia and lymphopenia, and this study mainly focused on LY. The total amount of peripheral blood LY is one of the most important indicators of immune activity. LY plays a central role in antitumor immunity. A low LY before treatment is often considered a poor prognostic factor for OS, recurrence, or metastasis in solid tumors (50, 51). Our results suggest that a high peripheral blood LY is significantly associated with MPR after NCIO and can be used as a predictor of whether patients will achieve MPR after NCIO. This may be due to a decrease in LY, reflecting reduced levels of TIL in the tumor microenvironment and an impaired cell-mediated immune response, which provides a favorable environment for tumor cell growth (52, 53). Some studies have also suggested that pre-treatment peripheral blood LY is positively associated with patients’ risk of immune-related adverse reactions (irAEs) (54). Because peripheral blood biomarkers are readily available and inexpensive, further studies are required to investigate them.

As a distinctive NSCLC histological subtype, LUSC has specific clinicopathological features, including older age, advanced disease at diagnosis, comorbidities, and central tumor location. Due to the tumor’s central location, tumor-associated pulmonary atelectasis is often observed. The current TNM staging system considers pulmonary atelectasis a poor prognostic factor (55). However, the prognostic value of pulmonary atelectasis is controversial. A previous study suggested that pulmonary atelectasis may prolong survival (56); however, according to Chen et al., pulmonary atelectasis had no discernible impact on the prognosis of patients with superficial bronchial lung cancer (57). Our study results showed that pulmonary atelectasis unfavorably influenced the MPR after NCIO. Patients with pulmonary atelectasis may not respond well to NCIO because of local immune dysfunction, dysregulated cytokine secretion, impaired immune cell and surfactant function due to hypoxia, lack of cyclic stretching, and inflammatory cell infiltration due to pulmonary atelectasis (58).

The results of this study suggest that the level of PD-L1 expression in tumor tissues correlates with the pathological remission of resected tumor tissues up to the MPR after surgery. Similarly, the classic neoadjuvant immunotherapy clinical studies mentioned earlier, including NADIM (17), CheckMate816 (16), SAKK 16/14 (59), and NEOSTAR (15), confirmed a positive correlation between PD-L1 expression and pathological response. Data from the last decade show that among NSCLC patients with high PD-L1 expression, the 5-year overall survival rate exceeds 25% (5). In contrast, studies such as CheckMate 159 (34) and LCMC3 (41) revealed that patients’ D-L1 expression levels were not linked with MPR and that patients benefited from neoadjuvant immune therapy. Another large meta-analysis based on 66 studies showed that the application of NCIO in NSCLC patients with PD-L1 expression levels ≥1% improved the pathological response rates and PFS/OS (60). The heterogeneity observed in previous studies may be related to the level of PD-L1 expression or its interaction with other factors, suggesting that the application of PD-L1 is somewhat limited, and further studies are needed to exclude other confounding clinical factors. However, the correlation between PD-L1 expression and immunotherapy efficacy has been widely recognized in clinical practice. Therefore, the relationship between PD-L1 and MPR/pCR needs to be further explored in future clinical trials so that the results of PD-L1 detection can help in the clinical screening of patients who can benefit from NCIO.

The biological properties of the original tumor and conventional TNM staging have been linked in some studies; in particular, the prognosis of the original tumor is closely linked to the lymph node (LN) status. For example, one investigator screened 109,026 NSCLC patients using the Surveillance, Epidemiology, and End Results Program (SEER) database and confirmed that the number or ratio of LNs in patients with NSCLC was an independent indicator of survival (61). In the NADIM study, the percentage of pCR in N2 patients was 55.88% (19/34), but in N0-1 patients, it was 58.33% (7/12) (17). The CheckMate-159 study showed that patients with LN metastases (+) who achieved the MPR were significantly fewer than those with LN (–) metastases (11.11% vs. 72.73%, respectively) (13). In the current study, the probability of achieving the MPR was 82.14% (23/28) for patients with N0-1 and 57.14% (36/63) for patients with N2-3, in agreement with the results of previous studies. By altering the function of immune cells, the intensification of LN metastasis encourages the local and systemic immunosuppression of cancer cells during the progression and metastasis of the primary tumor (62, 63), indicating that N2/3 LN metastasis signifies a greater growth of the immune escape mechanisms. The results of several other studies have shown that N2/N3 LN metastasis is significantly associated with adverse effects in patients treated with NCIO (64). Therefore, we postulated that this finding may be related to the immunosuppression and impaired immune function caused by N2/N3 LN metastasis.

However, this study has some limitations. First, this was a single center, retrospective study with a small sample size in which all patients were treated with surgery. However, in real clinical settings, some patients would not undergo surgery or complete NCIO because of unsatisfactory NCIO results. This difference, the lack of diversity, and the lack of patients overall may affect the generalizability of the results. Thus, in future studies, a large-scale multicenter prospective trial should be performed to explore which patients with LUSC may benefit from NCIO. Second, we could not exclude some LUSC patients with driver mutations because common mutations like EGFR and ALK are generally insensitive to immunotherapy. Third, this study did not focus on safety issues associated with NCIO, such issues could majorly impact the effectiveness of the strategy and patient/clinician decisions. Fourth, the PFS and OS data have not yet matured due to the short follow-up period, we need to continue long-term follow-up. Therefore, the findings of this study need to be validated and potential other mechanisms should be explored via long-term studies with large sample sizes.

## Conclusion

Our study demonstrated that NCIO had a high MPR rate in patients with stage IIB–IIIC LUSC. The independent predictors of prognosis were the peripheral blood LY, tumor burden, N classification, radiographic response, pulmonary atelectasis, and pre-treatment PD-L1 expression levels. A clinical prediction model was created using the above factors to screen suitable patients for NCIO. These findings suggest that these clinical factors could be used to assess NCIO suitability and prognosis in a clinical setting, reducing the number of unneeded procedures. Furthermore, the nomogram may be a useful tool for assuring customized therapy for patients with possibly resectable LUSC. In future studies, a large-scale multicenter prospective trial should be performed to further explore which patients with LUSC may benefit from NCIO.

## Data availability statement

The original contributions presented in the study are included in the article/supplementary material. Further inquiries can be directed to the corresponding author.

## Ethics statement

The ethics committees approved the retrospective protocol of the current study of Liaoning Cancer Hospital, which was conducted in accordance with the Declaration of Helsinki. Since all data are anonymous, the requirement of informed consent is exempted.

## Author contributions

YeW: Data curation, Formal analysis, Investigation, Methodology, Project administration, Resources, Software, Writing – original draft. YS: Software, Writing – review & editing. RW: Writing – review & editing. YuW: Writing – review & editing. ML: Writing – review & editing. KX: Writing – review & editing. RH: Writing – review & editing. ZW: Writing – review & editing. QL: Writing – review & editing. F-MK: Writing – review & editing. TW: Conceptualization, Data curation, Investigation, Methodology, Project administration, Resources, Supervision, Validation, Writing – original draft, Writing – review & editing.
